# A review of the process of knowledge transfer and use of evidence in reproductive and child health in Ghana

**DOI:** 10.1186/s12961-018-0350-9

**Published:** 2018-08-03

**Authors:** Gordon Abekah-Nkrumah, Sombié Issiaka, Lokossou Virgil, Johnson Ermel

**Affiliations:** 1 Department of Public Administration and Health Services Management, University of Ghana Business School, P. O. Box 72, Legon, Accra Ghana; 2 West African Health Organisation, 01 BP 153, Bobo-Dioulasso 01, Burkina Faso

**Keywords:** Knowledge transfer, Evidence, Reproductive and child health

## Abstract

**Background:**

The paper carries out a situational analysis to examine the production, dissemination and utilisation of reproductive and child health-related evidence to inform policy formulation in Ghana’s health sector.

**Methods:**

The study used Wald’s model of knowledge production, transfer and utilisation as a conceptual model to collect relevant data via interviews and administration of questionnaire to a network of persons who either previously or currently hold policy-relevant positions in Ghana’s health sector. Additional data was also gathered through a scoping review of the knowledge transfer and research utilisation literature, existing reproductive and child health policies, protocols and guidelines and information available on the websites of relevant institutions in Ghana’s health sector.

**Results:**

The findings of the study suggest that the health sector in Ghana has major strengths (strong knowledge production capacity, a positive environment for the promotion of evidence-informed policy) and opportunities (access to major donors who have the resources to fund good quality research and access to both local and international networks for collaborative research). What remains a challenge, however, is the absence of a robust institutional-wide mechanism for collating research needs and communicating these to researchers, communicating research findings in forms that are friendlier to policy-makers and the inability to incorporate funding for research into the budget of the health sector.

**Conclusion:**

The study concludes, admonishing the Ministry of Health and its agencies to leverage on the existing strengths and opportunities to address the identified challenges.

## Background

Evidence-based practice or evidence-informed policy-making generally refers to systematic efforts to ensure that research evidence becomes an important input into policy-making [1]. This has variously been referred to as knowledge translation, knowledge transfer, knowledge exchange, research utilisation, implementation, diffusion and dissemination [2]. Evidence-informed policy-making has assumed increased importance in several arenas of policy-making [3, 4]. In the area of health policy, the weight placed on evidence-informed policy is even much greater, with the reason that it leads to optimal allocation and fair distribution of resources, responds to scientific and technological advances and consequently improves health outcomes [5]. Indeed, there are a couple of high profile documents that have emphasised the importance of evidence-based policy/knowledge transfer in the healthcare arena. For example, WHO and the Lord Darzi report on England National Health Service have all emphasised the need for closer collaboration between users and producers of evidence to ensure that practice is evidence informed [6].

The idea that virtually all forms of policy should be based on strong scientific evidence can be traced to the establishment of the evidence-based medicine (EBM) framework. At the core of EBM is the use of clinical evidence (resulting from scientific research) to guide clinical practice. The growth of EBM has transcended clinical practice and greatly influenced the call for non-clinicians (policy-makers, government officials and programme managers) to abandon policy development approaches that rely heavily on common sense, popular support and political ideology in favour of approaches that are primarily based on scientific facts/evidence generated through appropriate and robust scientific research. It is not uncommon for discussions about evidence-informed policy to generate debate about what constitutes evidence. Generally, evidence can be operationalised to mean facts (actual or asserted) gained through observation or experiences and used to support a conclusion [7]. The National Institute for Health and Clinical Excellence (NICE) further argues that evidence can either be scientific or colloquial [8]. According to NICE, scientific evidence arises from explicit (codified and propositional), systematic (use of transparent and unambiguous methods for codification), and replicable (use of methods that can reproduce results in similar circumstances) scientific methods. On the contrary, colloquial evidence arises from expert testimony or comments from practitioners and stakeholders that may be crucial in complementing scientific evidence. Within the innovation literature, evidence is also argued to include experiences or received wisdom of individuals [9]. It is important however to emphasise that, among the different facts used to support a policy or conclusion, the most reliable is argued to be scientific evidence [10–12]. It is therefore not surprising that stronger health systems around the world (both developed and developing) are believed to be those whose health policies are informed by high quality scientific research evidence [1, 5].

Notwithstanding the importance of using scientific research evidence to guide the formulation and implementation of health policies, there is evidence in the research utilisation literature to suggest the existence of a major gap between available research evidence and actual practice (i.e. health policy and clinical practice) [13]. The research evidence to utilisation gap is much worse in low- to middle-income countries, partly due to weak capacity (skill set and systems) to carry out policy-relevant research and ability to translate research findings into a form that can be easily utilised by policy-makers [14]. Besides the issues of capacity, the underdeveloped nature of health systems in low- to middle-income countries such as Ghana, coupled with the generally low levels of investments in health, may also be responsible. At the micro level, it is equally argued that the propensity of healthcare organisations to use research evidence in policy is determined by their ability to put in place formal and informational structures that drives organisational learning and norms, and value the importance of evidence in decision-making [15–19]. The micro and macro level weaknesses enumerated above may possibly explain the low utilisation of research evidence in health policy-making in many developing countries.

Although Ghana’s investment in health has improved over the years and is regarded as one of the best in the sub-Saharan African region, it compares unfavourably to other developing countries. For example, health expenditure (HE) as a percentage of Gross Domestic Product (GDP) and HE per capita increased from 3% in 2000 to 5.4% in 2013 and USD 82.4 in 2000 to USD 214.2 in 2013, respectively. Ghana’s HE as a percentage of GDP and HE per capita in 2013 compares favourably to Kenya (4.5% and USD 44.5), Nigeria (3.5 and USD 115), The Gambia (6% and USD 28.9), Uganda (9.8 and USD 59.1) and Mali (7.1 and USD 53.3). Although Rwanda’s HE as a percentage of GDP is 2.5 percentage points higher than Ghana, their HE per capita of USD 83 is lower compared to Ghana [20].

Ghana’s health sector has witnessed appreciable progress in several areas over the last one and half decades. Although Ghana did not meet the Millennium Development Goals target on maternal and child health (i.e. MDGs 4 and 5), key outcome indicators in the area of reproductive and child health (RCH) have improved, such as a reduction in the national maternal mortality rate of 49%, from 760/100,000 live births in 1990 to 380/100,000 live births in 2013 [21]. Indeed, Ghana’s 2013 maternal mortality rate can be considered very low compared to that of neighbouring or other African countries such as Nigeria (560/100,000), Niger (630/100,000) and Sierra Leone (1100/100,000) over the same period [21]. Consumption of reproductive health inputs has also improved tremendously. The report of the 2014 Ghana Demographic and Health Survey (GDHS) suggested that the percentage of women receiving antenatal care from a skilled provider increased from 82% in 1988 to 97% in 2014, with the attendance of four or more antenatal visits also increasing from 78% in 2008 to 87% in 2014 [22]. In addition, 78% of women having given birth in the 5 years preceding the 2014 GDHS received protection against neonatal tetanus, with women delivering in a health facility increasing from 42% in 1988 to 73% in 2014. The GDHS 2014 report also suggested that 8 in 10 mothers received postnatal check-up within the crucial first 2 days after delivery.

Infant and child health has also improved over the years. For example, infant mortality and under-five mortality have declined by 28% and 44%, respectively, for the period 1998 to 2014. Additionally, the 2014 GDHS suggested that neonatal mortality and perinatal mortality stood at 29/1000 and 38/1000 live births, respectively, and that the percentage of children with basic vaccination coverage increased from 47% in 1998 to 77% in 2014. Although the percentage of children (aged 12–23 months) with low birth weight (less than 2.5 kg) is 10%, children under-five who are stunted, wasted or underweight dropped from 35%, 8% and 18%, respectively, in 2003 to 19%, 5% and 11%, respectively, in 2014 [22].

The substantial progress made in Ghana’s health sector has often been linked to the adoption of sector-wide approaches in the 1990s, which ushered in systematic approaches to policy formulation and implementation in Ghana’s health sector [23–25]. For example, the formulation and implementation of comprehensive and robust medium-term plans (i.e. the 5-Year Programme of Works (POWs)), starting from 1997 to date, are all products of the Ghana sector-wide approaches. There have been four 5-Year POWs (i.e. 1997–2001, 2002–2006, 2007–2011 and 2014–2017) since 1997. Most importantly, the POWs meant the formulation of programme-specific policies (for example, RCH) to achieve the objectives of the POWs at the national level.

What is clear and unambiguous about the POWs and other programme-specific policies (i.e. RCH policies) in the health sector is the clear, transparent, participatory and robust processes used in their development. On the contrary, the extent to which these policies are informed by existing scientific research evidence is either unclear or has rarely featured in Ghana’s health policy literature. The current paper therefore carries out a situational analysis to examine the process of knowledge production, transfer and use of scientific research evidence in the formulation of RCH policies in Ghana. For the purposes of this paper, RCH is defined to cover maternal, newborn and child health (MNCH).

## Conceptual model

The knowledge transfer and innovation diffusion literature abounds in several theoretical frameworks that aim to explain the process of knowledge transfer and eventual use of knowledge [14, 26–28]. For example, Roger’s Diffusion of Innovations theory has been used extensively in the last 20 years to explain the process of transferring knowledge into practice, especially in clinical practice, healthcare organisations and in health policy-making [29]. In addition, several authors, through systematic reviews of the existing knowledge transfer and research utilisation literature [30–36], have developed conceptual frameworks meant to explain the process of knowledge transfer and utilisation of research evidence in decision-making. Evidence from recent systematic reviews suggest the existence of as many as 63 different theoretical models and frameworks on knowledge transfer from fields such as healthcare, social care and management [26, 37]. There is also a section of the knowledge transfer/research utilisation literature that has focused on examining factors (knowledge characteristics, organisational characteristics, environmental characteristics, individual characteristics, etc.) that predict, facilitate or hinder the utilisation of research evidence [6, 28, 38]. Notwithstanding the abundance of theories and frameworks on knowledge transfer and research utilisation in this literature, the diverse nature and sheer depth of this literature makes it extremely difficult for practitioners and researchers alike to determine what constitutes an appropriate model [39]. Besides, several of these models are mostly unrefined and untested, thereby raising justifiable questions about their suitability for use in the design and evaluation of knowledge transfer interventions or understanding the research utilisation process [6]. A key criticism of most of the existing models is that they are very narrow and hardly cover the broader sociological processes in knowledge transfer [6].

**Fig. 1:**
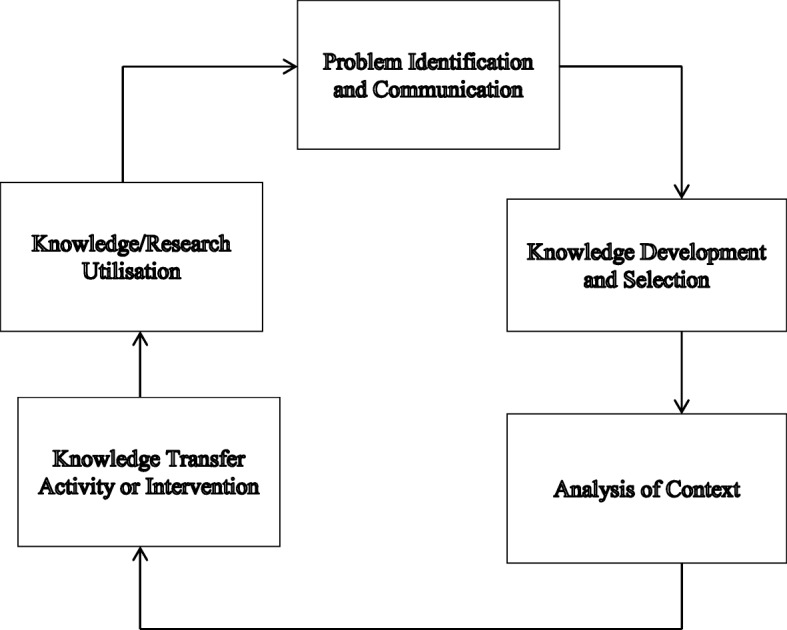
Knowledge Transfer and Research Utilisation Framework (KTRUF). Source: Constructed based on Ward et al. [6]

To better understand the process of knowledge transfer and use of evidence in decision-making, a framework that captures the broader sociological explanation of knowledge transfer and use of research evidence has recently been developed based on a comprehensive systematic review of the existing knowledge transfer literature [6]. Although the framework has not been empirically tested just as a large chunk of those before it, its appeal arises due to the fact that it is recent and tends to combine the components of 28 different models that either wholly or partly explains the process of knowledge transfer [6]. The framework is made up of five components as explained below (Fig. 1).

- Problem identification and communication – This deals with channels used by users to communicate problems to researchers.
- Knowledge/research development and selection – This deals with the knowledge or research to be transferred and attributes or characteristics that will enhance successful transfer of knowledge. Within the literature, key activities considered to be crucial at this stage of the knowledge transfer process include producing, synthesising and adapting to new knowledge.
- Analysis of context – This is part of the knowledge transfer process and looks at factors that may constrain or promote the transfer of knowledge.
- The knowledge transfer activity or intervention – This is often the most common component of the knowledge transfer process and involves the actual activities undertaken to transfer knowledge.
- Knowledge/research utilisation – This looks at the actual use of knowledge or research findings transferred.

The order in which the components of the framework are listed does not in any way suggest that they happen in a linear fashion. The proponents of the framework argue that knowledge transfer does not happen in a linear fashion, but in a complex, dynamic and multidirectional fashion [6]. This view has support in the existing literature, in that researcher to policy-maker interactions constitute a key ingredient to successful knowledge transfer [40, 41]. However, for simplicity and application to RCH in Ghana, our analysis will be conducted as if the process occurs in a linear form. Thus, the above five-component framework will constitute the basis for examining processes for the production, transfer and use of research evidence to inform RCH policy formulation in Ghana.

## Methods

Using a qualitative research method, the paper conducts a situational analysis of the process of knowledge transfer and utilisation of research evidence to inform RCH policy in Ghana. Data for the paper was gathered through a two-stage process. The first stage focused on collecting initial data for analysis. The first step of the first stage reviewed grey literature (i.e. policies, protocols and practice guidelines; Tables 1 and 2) that could potentially provide information on the use of evidence to inform policy. This was followed with interviews and discussions with persons who either previously held or currently hold a senior level position (Director, Deputy Director, Programme Officer, etc.) in the GHS or Ministry of Health (MOH). The discussions and interviews focused on understanding the capacity of the health sector to produce, disseminate and use research evidence to inform RCH policy.

**Table 1:** Profile and characteristics of policy documents on reproductive and child health in Ghana

S/No	Year of publication	Title of policy	Document source/publisher	Main focus of policy	Main research/evidence/ publication informing the policy cited in document	Process employed in the policy
1.	Ghana Health Service (GHS), 2003 [55]	National Breastfeeding Policy	GHS, Accra	Improve upon maternal and child health through the promotion, protection and support of optimal breastfeeding practices and appropriate complementary feeding practices	No scientific evidence was cited in the document, but other views of professionals were reflected	Developed through expert views
2.	Ministry of Health, 2014 [56]	Ghana National Newborn Health Strategy and Action Plan 2014–2018	Ministry of Health (MOH), Accra-Ghana	Provides a newborn scale-up strategy and action plan for addressing newborn mortality in Ghana	Employed a scientific evidence-based process for policy development	Adopted a detailed desk review of relevant policy on maternal and child health system performance in Ghana. Data on Household Survey (DHS, Multiple Indicator Cluster Surveys) were reviewed. Field visit was adopted, stakeholders discussions were initiated
3.	Ministry of Health, 2007 [57]	Under 5 child Health Policy: 2007–2015	MOH, Ghana	Provides a framework for programme planning, implementation and evaluation geared towards improving child survival and wellbeing. Provides standards and guidelines to prevention and treatment of child illness	No academic publication was cited in the document	Developed through consultation of stakeholders’ views and a review of relevant policy documents
4	Ghana Health Service, 2015 [58]	Report on the Burden of Obstetric Fistula in Ghana	GHS, Ghana UNFPA	Report on the burden of obstetric fistula in Ghana, examining personnel capacity in managing the condition	Employed scientific research evidence in the document write-up	Adopted a qualitative research approach with a survey in document preparation
5	Ministry of Health, 2013 [59]	Postpartum Hemorrhage Prevention and Management Strategy for Ghana	MOH, Accra PATH	Provides data on the incidence of postpartum haemorrhage in Ghana. Establishes a framework and guideline for postpartum haemorrhage prevention and management	Cited some empirical-based research	Collection of expert views and stakeholder consultation as well as a review of some relevant institutional policy, reports and household survey
6	Ministry of Health, 2007 [60]	Anti-Malaria Drug policy for Ghana	MOH, Accra	Provides policy measures and guidelines for the treatment of Malaria in Ghana	No publication is cited	Developed through stakeholder consultation and expert views
7	Ministry of Health, 2015 [61]	National Condom and Lubricant Strategy 2016–2020	Ghana AIDS Commission, Accra USAID UNFPA GHS, Accra	Establishes a national strategic framework to promote quality sexual and reproductive health	No publication was cited in the document	Collection of information from national strategic and action plan for HIV/AIDS prevention, reproductive health, etc. There was a consultation with other stakeholders

**Table 2:** Profile and characteristic protocols and practice guidelines on reproductive and child health in Ghana

S/No	Year of publication	Title of policy	Document source/publisher	Main focus of policy	Main research/evidence publication informing the policy cited in document	Process employed in the policy
1	Ghana Health Service, 2000 [62]	National Family Planning Protocols	Ghana Health Service (GHS), Accra	Aims at providing a strategic guide to information on family planning methods in Ghana	No empirical evidence was cited in the document	Document was based on expert views and standards of operating procedure by professionals
2	Ghana Health Service, 2008 [63]	National Safe Motherhood Service Protocol	GHS, Accra Ministry of Health (MOH), Accra	Provides a guideline for treating and managing pregnancy-related complications common to caregivers at all levels	No empirical evidence was cited in the document	Based on the views and standard operating procedure by experts in the field
3	Ghana Health Service, 2007 [64]	Adolescent Health Info Pack	GHS, Accra UNFPA UKaid	Focuses on the growing changes of adolescent (biological and social changes) Provides a guide on the risky sexual behaviours at the adolescent stage	No publication cited in the document	Developed from expert views and role play by adolescents to depict growing changes in the adolescent stage
4	Ministry of Health, 2011 [65]	What Every Pregnant Woman Should Know	MOH, Ghana	Provides information on the expectation of every pregnant woman based on the stages of pregnancy	No empirical evidence cited in the document	Based on the views and professional experiences of experts
5	UNFPA, 2014 [66]	Living with Fistula	UNFPA	Explore the views and lives of fistula survivors	No publication was cited in the document	Collection based on survivors’ views, experiences and funding partners support to survivors
6	Ministry of Health, 2010 [67]	Concise Integrated Management of Neonatal and Childhood Illness	MOH, Accra	Provides a strategy for managing childhood diseases and a guide for preventing cause of death	No publication was cited in the document	Based on expert clinical opinion and expert survey in the field of neonatal and child health
7	Ghana Health Service, 2010 [68]	Maternal Health & Death Audit Guidelines	GHS, Accra	Aims at providing a framework for improving maternal health quality and a tool for monitoring maternal deaths in Ghana	No empirical evidence was cited; however, an institutional policy document was cited	Employed a household survey method with expert views and a review of relevant policy documents
8	Ghana Statistical Service, 2007 [69]	Ghana Maternal Health Survey	GHS, GSS Inc. Macro	Provides empirical evidence on the incidence of maternal mortality. Establishes the prevalence of abortion in Ghana and provides guidelines on antenatal care attendance in Ghana	Empirical scientific evidence was cited in the document	Developed based on a household survey, review of relevant policy document and stakeholder involvement in the preparation of this document
9	Ghana Health Service [70]	Ghana Health Service Newsletter for Adolescent and Young People	GHS, Accra	Focuses on providing adolescent with information on adolescence-related challenges	No scientific evidence was established in the preparation of this document	Developed based on view of adolescent and stakeholder consultation
10	Ministry of Health, 2014 [68]	Guideline for Case Management of Malaria in Ghana	MOH, Accra	This policy is focused on providing guidelines for malaria case management in Ghana	No empirical evidence is cited in document preparation	Produced through expert views and professional knowledge on the management of malaria in Ghana
11	Ministry of Health, 2015 [71]	National Policy and Guidelines for Infection Prevention and Control in Health Care Settings	MOH, Ghana	Lays down policies and broad guidelines required for the practice of a nationally acceptable standard of infection prevention and control in healthcare settings	Document cites several scientific research papers and laws of Ghana as well as reports from key international organisations	Through a consultative approach, including several government and non-governmental organisations and individuals
12	Ghana Health Service, 2014 [72]	National Reproductive Health Service Policy and Standards, 3rd Edition	GHS, Accra	Provides explicit directions and focus for streamlining the training and service provision of reproductive health in addition to programmes that make reproductive health accessible and affordable	No scientific research paper was cited; however, relevant laws in Ghana related to abortion and crime were also cited	A consultative approach including government agencies, regulatory bodies, development partners, NGOs and other champions working in the area of reproductive health
13	Ghana Health Service, 2012 [73]	Prevention and Management of Unsafe Abortion: Comprehensive Abortion Care and Services Standards & Protocols	GHS, Accra	Provision of critical guide for the prevention and management of unsafe abortion	Document cite several scientific research papers as well as documents from other key research organisations	Prepared jointly, and in different stages, by the GHS/MOH and several institutions, individuals and communities
14	World Health Organization, 2010 [74]	Adolescent Job Aid: A Handy Desk Reference Tool for Primary Level Health Workers	WHO	It contains guidance on commonly occurring adolescent-specific or non-adolescent-specific problems or concerns that have not been addressed in existing WHO guidelines, conditions in adolescents	No scientific research evidence was cited in the document	Evidence-based approach together with extensive consultation and country level testing for further evidence was used in developing the document
15	Ministry of Health, 2010 [75]	Malaria in Pregnancy. Training for Providers	MOH, Accra USAID, WHO, GLOBAL FUND	Provides strategic guide to health providers for malaria treatment in Ghana	No publication was cited	Prepared through role play, case studies and expert views

Additional data was also acquired through the review of information provided on the website of MOH and its agencies, specifically, GHS and related departments. These include the Navrongo Health Research Centre (NHRC), the Kintampo Health Research Centre (KHRC) and the Dodowa Health Research Centre (DHRC). The website review focused on gathering information on the capacity of the research centres to produce evidence and convert the evidence to a form that can easily be used by policy-makers. In addition, the websites of the GHS research centres (https://mamaye.org/welcome-e4a-mamaye), a popular website with informative content on RCH issues in Ghana and other West African countries was also reviewed. Additionally, RCH-related literature on Ghana was reviewed to gather evidence on the capacity of Ghana’s health sector to generate scientific evidence to support policy-making in RCH. This was done via a search through recognised public health-related databases and publishers (BMC, Elsevier, Oxford, PubMed, African Journals Online and Global Health Archives) and Google Scholar using the following search topics: (1) knowledge transfer and health policy; (2) evidence-informed health policy; (3) evidence and maternal health policy in Ghana; (4) evidence and child health policy in Ghana; (5) evidence and newborn health policy in Ghana; and (6) reproductive and child health intervention scale-up in Ghana.

Based on the above search criteria, peer-reviewed journal articles on RCH published in English since 2000, were retrieved. Overall, 77 out of a total of 534 articles retrieved were selected and reviewed. The 77 were selected on the basis of relevance and are made up of (1) 39 articles on RCH-related evidence in Ghana with at least one author being a staff member of MOH/GHS (Table 3); (2) 28 articles on RCH-related evidence in Ghana authored by researchers outside of MOH/GHS (Table 4); and (3) 10 articles on scaling-up of RCH-related projects in Ghana (Table 5).

**Table 3:** Profile and characteristics of scientific contributions of staff from the Ghana Ministry of Health/Ghana Health Service and related agencies

	Authors	Author of interest	Title of paper	Place of work	Name of Journal
1.	Ganyaglo and Hill, 2012 [76]	Gabriel YK Ganyaglo	A 6-Year (2004–2009) Review Of Maternal Mortality at the Eastern Regional Hospital, Koforidua, Ghana	Department of Obstetrics and Gynaecology, Korle Bu Teaching Hospital, Korle-Bu, Accra, Ghana	*Seminars in Perinatology*
2.	Orish et al., 2012 [77]	Verner N Orish	Adolescent Pregnancy and the Risk of *Plasmodium falciparum* Malaria and Anaemia – A Pilot Study from Sekondi-Takoradi Metropolis, Ghana	Department of Internal Medicine, Effia-Nkwanta Regional Hospital Sekondi-Takoradi, Sekondi, Western Region, Ghana	*Acta Tropica*
3.	Ö Tunçalp et al., 2013 [78]	Kwame Adu-Bonsaffoh	Assessment of Maternal Near-Miss and Quality of Care in a Hospital-Based Study in Accra, Ghana	Department of Obstetrics and Gynaecology, Korle-Bu Teaching Hospital, Accra, Ghana	*International Journal of Gynecology and Obstetrics*
4.	Abdullah et al., 2011 [79]	Francis Abantanga, Elias Sory Hayley Osen	Assessment of Surgical and Obstetrical Care at 10 District Hospitals in Ghana Using On-site Interviews	Department of Surgery, Korle Bu Teaching Hospital, Accra, Ghana Department of Surgery, Komfo Anokye Teaching Hospital, Kumasi, Ghana Ghana Health Services, Accra, Ghana	*Journal of Surgical Research*
5.	Asundep et al., 2013 [80]	Cornelius Archer Turpin	Determinants of Access to Antenatal Care and Birth Outcomes in Kumasi, Ghana	Komfo Anokye Teaching Hospital, Kumasi, Ghana	*Journal of Epidemiology and Global Health*
6.	Kirkwood et al., 2010 [48]	S Amenga-Etego C Tawiah C Zandoh, S Danso S Owusu-Agyei, P Arthur	Effect of Vitamin A Supplementation in Women of Reproductive Age on Maternal Survival in Ghana (ObaapaVitA): A Cluster-Randomised, Placebo-Controlled Trial	Kintampo Health Research Centre, Ministry of Health, Kintampo, Ghana	*Lancet*
7.	Dassah et al., 2015 [81]	Edward T. Dassah Yaw Adu-Sarkodie	Estimating the Uptake of Maternal Syphilis Screening and Other Antenatal Interventions before and after National Rollout of Syphilis Point-of-Care Testing in Ghana	Department of Obstetrics and Gynecology, Komfo Anokye Teaching Hospital, Kumasi, Ghana	*International Journal of Gynecology and Obstetrics*
8.	Rominski et al., 2014 [82]	1. Raymond Aborigo 2. Abraham Hodgson	Female Autonomy and Reported Abortion-Seeking in Ghana, West Africa	Navrongo Health Research Centre, Ghana Health Service, Navrongo, Ghana Ghana Health Service, Accra, Ghana	*International Journal of Gynecology and Obstetrics*
9.	Hussein et al., 2005 [83]	Mercy Abbey	How do Women Identify Health Professionals at Birth in Ghana?	Ghana Health Service, Health Research Unit, Accra, Ghana	*Midwifery*
10.	Geynisman et al., 2011 [84]	Anthony Ofosu	Improving Maternal Mortality Reporting at the Community Level with a 4-Question Modified Reproductive Age Mortality Survey (RAMOS)	Ghana Health Service and Ministry of Health, Accra, Ghana	*International Journal of Gynecology and Obstetrics*
11.	Morhe et al., 2012 [85]	Emmanuel S.K. Morhe Frank K. Ankobea Kwabena A. Danso	Reproductive Experiences of Teenagers in the Ejisu-Juabeng District of Ghana.	Department of Obstetrics and Gynecology, Komfo Anokye Teaching Hospital, Kumasi, Ghana	*International Journal of Gynecology and Obstetrics*
12.	Okiwelu et al., 2007 [86]	Daniel Arhinful Margaret Armar-Klemesu	Safe Motherhood in Ghana: Still on the Agenda?	Noguchi Memorial Institute for Medical Research, University of Ghana, Accra, Ghana	*Health Policy*
13.	Witter et al., 2007 [87]	Sawudatu Zakariah-Akoto	The Experience of Ghana in Implementing a User Fee Exemption Policy to Provide Free Delivery Care	Researcher, IMMPACT, Noguchi Memorial Institute of Medical Research, University of Ghana	*Reproductive Health Matters*
14.	Subramanian et al., 2010 [88]	Nicholas Kanlisi	The Ghana Vasectomy Initiative: Facilitating Client–Provider Communication on No-scalpel Vasectomy	Ghana R3M Project, Engender Health, Accra, Ghana	*Patient Education and Counseling*
15.	Ako and Akweongo, 2009 [89]	Matilda Aberese Ako, Patricia Akweongob	The Limited Effectiveness of Legislation Against Female Genital Mutilation and the Role of Community Beliefs in Upper East Region, Ghana	Research Fellow, Navrongo Health Research Centre, Navrongo, Upper East Region, Ghana	*Reproductive Health Matters*
16.	Moyer et al., 2014 [90]	Raymond A. Aborigo Abraham Hodgson,	‘They Treat you like you are not a Human Being’: Maltreatment During Labour and Delivery in Rural Northern Ghana	Navrongo Health Research Centre, Navrongo, Upper East Region, Ghana	*Midwifery*
17.	Masters et al., 2013 [91]	1. George Amofah 2. Patrick Abaogye	Travel Time to Maternity Care and its Effect on Utilization in Rural Ghana: A Multilevel Analysis	Ghana Health Service, Kumasi, Ghana Reproductive and Child Health Dept., Family Health Division, Ghana Health Service, Accra, Ghana	*Social Science & Medicine*
18.	Baiden et al., 2006 [92]	Baiden F, Amponsa-Achiano K, Oduro AR, Mensah TA, Baiden R, Hodgson A	Unmet Need for Essential Obstetric Services in a Rural District in Northern Ghana: Complications of Unsafe Abortions Remain a Major Cause of Mortality	1. Navrongo Health Research Centre, Navrongo, Upper East Region, Ghana 2. Department of Obstetrics and Gynaecology, Kwame Nkrumah University of Science and Technology, Kumasi, Ghana 3. War Memorial Hospital, Navrongo, Upper East Region, Ghana	*Public Health*
19.	Powell-Jackson et al., 2014 [93]	Evelyn K Ansah	Who Benefits from Free Healthcare? Evidence from a Randomized Experiment in Ghana	Research and Development Division, Ghana Health Service, Ghana	*Journal of Development Economics*
20.	Sinclair et al., 2013 [94]	Martha Gyansa-Lutterodt, Brian Asare, Augustina Koduah	Integrating Global and National Knowledge to Select Medicines for Children: The Ghana National Drugs Programme	Ghana National Drugs Programme, Accra, Ghana	*PLoS Medicine*
21.	Dassah et al., 2015 [95]	Edward Tieru Dassah, Yaw Adu-Sarkodie	Factors Associated with Failure to Screen for Syphilis During Antenatal Care in Ghana: A Case Control Study	Department of Obstetrics and Gynaecology, Komfo Anokye Teaching Hospital, Kumasi, Ghana	*BMC Infectious Diseases*
22.	Cofie et al., 2015 [96]	Sodzi Sodzi-Tettey	Birth Location Preferences of Mothers and Fathers in Rural Ghana: Implications for Pregnancy, Labor and Birth Outcomes	Project Fives Alive!/Institute for Healthcare Improvement, Accra, Ghana	*BMC Pregnancy and Childbirth*
23.	Atuahene et al., 2013 [97]	David Mensah and Martin Adjuik	A Cross-Sectional Study of Determinants of Birth Weight of Neonates in the Greater Accra Region of Ghana	National Malaria Control Programme, Accra, Ghana. 3INDEPTH Network Secretariat, Accra, Ghana	*Maternal Health, Neonatology, and Perinatology*
24.	Nakua et al., 2015 [98]	Justice Thomas Sevugu	Home Birth without Skilled Attendants Despite Millennium Villages Project Intervention in Ghana: Insight from a Survey of Women’s Perceptions of Skilled Obstetric Care	Sekyere-Kumawu Health Directorate, Kumasi, Ghana	*Pregnancy and Childbirth*
25.	Dalaba et al., 2015 [99]	Maxwell A Dalaba Raymond A Aborigo John Williams Gifty A Aninany	Cost to Households in Treating Maternal Complications in Northern Ghana: A Cross Sectional Study	Navrongo Health Research Centre, Navrongo, Ghana	*Health Services Research*
26.	Manu et al., 2015 [100]	Gloria Quansah Asare, Kwasi Odoi-Agyarko	Parent–Child Communication about Sexual and Reproductive Health: Evidence from the Brong Ahafo Region, Ghana	Family Health Division, Ghana Health Service, Private Mail Bag, Ministries, Accra, Ghana RHI Medical Centre, Amanokrom, Mampong-Akuapem, Eastern Region, Ghana	*Reproductive Health*
27.	Achana et al., 2015 [101]	Fabian Sebastian Achana, Paul Welaga, Timothy Awine, Abraham Oduro, John Koku Awoonor-Williams	Spatial and Socio-Demographic Determinants of Contraceptive use in the Upper East Region of Ghana	Navrongo Health Research Centre, Navrongo, Upper East Region, Ghana Regional Health Directorate, Ghana Health Service PMB, Upper East Region, Bolgatanga, Ghana	*Reproductive Health*
28.	Adjei et al., 2015 [102]	1. Kwame K. Adjei, Martha A. Abdulai, Sam Newton, and Seth Owusu-Agyei 2. Sam Adjei	A Comparative Study on the Availability of Modern Contraceptives in Public and Private Health Facilities in a Peri-Urban Community in Ghana	Kintampo Health Research Centre, Ghana Health Service, Kintampo, Ghana Centre for Health and Social Services, Accra, Ghana	*Reproductive Health*
29.	Amoakoh-Coleman et al., 2015 [103]	Charles Brown-Davies Kerstin Klipstein-Grobusch	Completeness and Accuracy of Data Transfer of Routine Maternal Health Services Data in the Greater Accra Region	Ghana Health Service, Greater Accra Region, Accra, Ghana Research and Development Division, Ghana Health Service, Accra, Ghana	*Research Notes*
30.	Hall et al., 2015 [104]	Kwabena Danso	A Retrospective Analysis of the Impact of an Obstetrician on Delivery and Care Outcomes at Four District Hospitals in Ghana	Ghana Health Service, Sekyere Kumawu District Health Directorate, Kumawu, Ghana	*International Journal of Gynecology and Obstetrics*
31.	Bawah et al., 2009 [105]	Ayaga Bawah	The Impact of Immunization on the Association between Poverty and Child Survival: Evidence from Kassena-Nankana District of Northern Ghana	The INDEPTH Network	*Scandinavian Journal of Public Health*
32.	Adongo et al., 1997 [106]	Adongo B. Philip	Cutural Factor Constraining the Introduction of Family Planning among the Kassena-Nankana District of Northern Ghana	Navrongo Health Research Centre, Ministry of Health, Navrongo, Ghana	*Social Science & Medicine*
33.	Amoakohene, 2004 [107]	Amoakohene	Violence against Women in Ghana: A Look at Women’s Perceptions and Review of Policy and Social Responses	Ghana Health Service	*Social Science & Medicine*
34.	Witter et al., 2009 [108]	Margaret Armar-Klemesu	Providing Free Maternal Health Care: Ten Lessons from an Evaluation of the National Delivery Exemption Policy in Ghana	Noguchi Memorial Institute for Medical Research, Accra, Ghana	*Ghana Global Health Action*
35.	Oppong et al., 2015 [109]	Samuel A. Oppong, Michael Y. Ntumy, Mary Amoakoh-Coleman, Deda Ogum-Alangea, Emefa Modey-Amoah	Gestational Diabetes Mellitus among Women Attending Prenatal Care at Korle-Bu Teaching Hospital, Accra, Ghana	Ghana Health Service, Accra, Ghana	*International Journal of Gynecology and Obstetrics*
36.	Obrist et al., 2014 [110]	Ernest Osei-Bonsu, Baffour Awuah	Factors Related to Incomplete Treatment of Breast Cancer in Kumasi, Ghana	Komfo Anokye Teaching Hospital, Department of Medical Oncology and Radiation, & Central Administration, Kumasi, Ghana	*Breast*
37.	Shelus et al., 2015 [111]	Stephen Mensah, Kafui Dzasi	Lessons from a Geospatial Analysis of Depot Medroxyprogesterone Acetate Sales by Licensed Chemical Sellers in Ghana	Global Health Population and Nutrition, Accra, Ghana	*International Journal of Gynecology and Obstetrics*
38.	Sukums et al., 2015 [112]	Nathan Mensah, Afua Williams	Promising Adoption of an Electronic Clinical Decision Support System for Antenatal and Intrapartum Care in Rural Primary Healthcare Facilities in Sub-Saharan Africa: The QUALMAT Experience	Navrongo Health Research Centre, Navrongo, Ghana	*International Journal of Medical Informatics*
39.	Aborigo et al., 2015 [113]	Akawire Aborigo	The Traditional Healer in Obstetric Care: A Persistent Wasted Opportunity in Maternal Health	Navrongo Health Research Centre, Navrongo, Ghana	*Social Science & Medicine*

**Table 4:** Profile and characteristics of available evidence outside of Ghana Ministry of Health/Ghana Health Service that can be used to inform reproductive and child health policy in Ghana

S/No	Author/year of publication/reference	Category of intervention	Health issue of intervention	Evidence generated	Policy-relevant conclusion
PAPERS ON MATERNAL HEALTH					
1.	de Souza, 2009 [114]	Evaluation of evidence-based approaches to decision-making	Inclusion of geographic information systems (GIS) as part of Ghana’s health information systems	GIS health applications in Ghana are few, with little or perhaps no effects on policy and decision-making Including GIS as part of Health Information Systems can go a long way in promoting the generation and use of evidence for decision-making, programme development, resource allocation and surveillance systems	A strong collaboration between academics in the area of GIS and health professionals in the Ghana Health Service will be key to advancing health-based GIS
2.	Awusabo-Asare et al., 2004 [115]	Research evidence on adolescent sexual and reproductive health	A synthesis of research evidence on adolescent sexual and reproductive health	Evidence suggest a sizeable gap between the age at first sex and the age at first marriage; generally, first sexual intercourse happens about 2 years before first marriage for young women, with young men taking place about 5 years before first marriage	Notwithstanding that young people are aware of the existence of different contraceptive methods, including male condoms, usage remains relatively low, with access to health information and services for young people being uneven
3.	Croce-Galis, 2004 [116]	Evidence on sexual and reproductive health	Documents what is known about Ghanaian adolescents’ sexual and reproductive health behaviours and needs, with particular emphasis on HIV/AIDS	There is a lack of information about the implementation, monitoring and, most importantly, the evaluation of interventions aimed at improving the sexual and reproductive health of Ghanaian youth	Additional evidence is required to explain the gap between awareness of sexual and reproductive health services and actual use of such services as well as information about the implementation, monitoring and, most importantly, the evaluation of interventions aimed at improving the sexual and reproductive health
4.	Baker et al., 2012 [117]	Promotion of maternal healthcare intervention, which is already operational	Understanding how to increase clinical practice guideline potential to improve quality of care for mothers in three sub-Saharan African countries including Ghana	The study suggests very little variance between national guidelines for maternal health and WHO recommendations; this is not withstanding the that use of clinical practice guidelines in practice was perceived to be limited	There is need to prioritise the format of guidelines to increase their usability and applicability and to consider these attributes together with implementation as integral to their development processes; the prioritisation of the format of guidelines will improve applicability and usability
5.	Mayhew, 2000 [118]	Evaluation of a range of policies developed for sexually transmitted infection (STI) management	Integrating STI services in family planning (FP)/maternal and child health (MCH) services	The paper suggests that a ‘blanket’ policy to integrate STI and FP/MCH services may be inappropriate in particular contexts; the implementation of health policies is influenced, and often impeded not only by local service contexts, economic and epidemiological factors, but also by culturally defined social attitudes and behaviours	Enhancing, at district level, the voice of nurses working at the community level and promoting collaborative, culturally specific and community-based initiatives could facilitate addressing the issues
6.	Ganyaglo and Hill, 2012 [76]	Review of issues on maternal mortality	A 6-year review of maternal mortality	The study revealed that the highest number of deaths was recorded in the period following termination of pregnancy (abortion or delivery)	Referral of patients to hospital on a timely basis could be important for reducing preventable maternal deaths
7.	Darteh et al., 2014 [119]	Decision-making in reproductive health (RH).	Examination of factors that influence the decision to engaging in sexual intercourse and condom use among women	Women who were in the richest, rich and middle wealth index quintiles were more likely to make decisions to engage in sexual intercourse as well as use condoms compared to the poorest; additionally, women with some level of education were more likely to make decisions on sexual intercourse than those with no formal education	Interventions and policies aimed at empowering women to take control of their reproductive health should target women from less wealthy backgrounds and those with low educational attainments
8.	Sundaram et al., 2014 [120]	Evaluation of MHC intervention already in operation	Examination of whether the R3M programme made a difference to the provision of safe abortion services and post abortion care; also examine the role played by provider attitudes and knowledge of the abortion law, and on providers with clinical training in service provision	Associations between provider attitudes and knowledge of the law on both outcomes were either non-significant or inconsistent, including for providers with clinical knowledge of abortion provision; provider confidence however is strongly associated with service provision	The R3M programme is important for safe abortion provision; increased provider confidence is crucial to improving both safe abortion provision and post-abortion care
9.	Reichenbach, 2002 [121]	Evaluation of the politics of priority-setting in RH	Examines the influence of political and organisational factors on national priority-setting for reproductive health	Traditional priority-setting methods cannot explain the priority given to breast cancer in Ghana; it demonstrates how local politics can trump scientific and economic evidence and suggests that the priority-setting process can have unforeseen equity and social implications	The policy priority measure provides a more complete picture of reproductive health priorities and is useful for better understanding of the implications of the priority-setting process for reproductive health
10.	Amoako et al., 2015 [122]	Evaluation of MHC intervention already in operation	Investigated the impact of maternity-related fee payment policies on the uptake of skilled birth care amongst the poor in Ghana	The rich–poor gap in skilled birth care use was highly pronounced during the ‘cash and carry’ and ‘free antenatal care’ policies period; the benefits during the ‘free delivery care’ and ‘NHIS’ policy periods accrued more for the rich than the poor	The maternal care fee exemption policies specifically targeted towards the poorest women had limited impact on the uptake of skilled birth care
11.	Twum-Danso et al., 2014 [123]	Evaluated MHC intervention already in operational	Test the feasibility and effectiveness of the new early post-natal care (PNC) policy and its subsequent scale-up throughout northern Ghana	There is a slower increase in skilled delivery over a longer period of time; the early PNC policy was scaled up over the subsequent 2 years to 576 health facilities in all 38 districts of northern Ghana	The study provides a model for improving the implementation of other national health policies to accelerate the achievement of the Millennium Development Goals in Ghana and other resource-poor countries
PAPERS ON CHILD HEALTH					
1	Friedman et al., 2015 [124]	Evaluated child healthcare (CH) intervention already in operation	Using SMS from licensed chemical sellers in Ghana to recommend the use of oral rehydration salts and zinc for the management of childhood diarrhoea	Using SMS intervention, providers self-reported practices improved but not their actual practices	Actual practices vary considerably from reported practices
2	Tawiah-Agyemang et al., 2008 [125]	Evaluated a CH intervention already operational	Probed the reason why women in Ghana initiate breast-feeding early or late, who advices on initiation and what foods or fluids are given to babies when breast-feeding initiation is late	Facilitating factors that aided for early inception included delivery in a health facility, where the staff encouraged early breast-feeding, and the belief in some ethnic groups that putting the baby to the breast encourages the flow of milk	Raising awareness on early initiation of breastfeeding in the communities and the policy arena is crucial with interventions focusing on finding solutions to barriers to early initiation with a community component
3	Edmond et al., 2007 [126]	Evaluated a CH intervention already operational	Looked at early infant feeding practices and its effect on infection-specific neonatal mortality in breastfed neonates aged 2–28 days	No clear associations were seen between these feeding practices and non-infection-specific mortality; prelacteal feeding was not associated with infection or non-infection-specific mortality	This study gives the first epidemiologic proof of a causal association between early breastfeeding and reduced infection-specific neonatal mortality in young human infants
4	Manu et al., 2014 [127]	Evaluated a CH intervention already implemented	Evaluation of community volunteer assessment and referral implemented within the Ghana Newhints home visits cluster-randomised controlled trial	In resource-constrained settings, community volunteers can be successfully used to identify through assessment and referral of sick newborns to health facilities as recommended in the WHO/UNICEF joint statement on home visits in 2009	Isolated community interventions will have limited impact unless coupled with concurrent improvement of quality within health facilities
5	Adongo et al., 2005 [128]	Use of health knowledge in policy	Explored how local community knowledge about malaria acts as a barrier to the use of insecticide-treated nets in three settings	People recognise the term ‘malaria’ but have limited biomedical knowledge of the disease, including its aetiology, the role of the vector, and host response; convulsions and anaemia are rarely linked to malaria	Simply informing communities that mosquitoes cause malaria does not appeal to people; health education needs to move beyond that and inform people why it is the mosquito that causes malaria and not other insects
6	Nyarko et al., 2001 [129]	Immunisation status and child survival	Examine the influence of immunisation coverage on all-cause child mortality in Kassena-Nankana District of northern Ghana	Children who have received no immunisations are at substantially higher risk of death through approximately the first year of life than those who have some vaccination coverage, whether complete or incomplete	There is the need for further research on the all-cause mortality impact associated with these vaccines in developing countries
7	Singh et al., 2013 [130]	Impact evaluation of MCH intervention already operational	Evaluate the influence of the early phase of Project Fives Alive!, a national child survival improvement project, on key maternal and child health outcomes	There was an association between the early pregnancy identification change category with increased skilled delivery; also, a greater number of change categories tested was associated with increased skilled delivery	The quality improvement approach of testing and implementing simple and low cost locally inspired changes has the potential to lead to improved health outcomes at scale both in Ghana and other low- and middle-income countries
8	Vance et al., 2014 [131]	Implemented a RH intervention to improve CH	Determined whether integrating FP messages and referrals into facility-based, child immunisation services increase contraceptive uptake in the 9- to 12-month post-partum period	Reported referrals to FP services did not improve nor did women’s knowledge of factors related to return of fecundity	Rigorous evidence of the success of integrated immunisation services in resource-poor settings remains weak
9	Lei et al., 2006 [132]	Implemented an intervention in CH	Assessed the effect of a millet drink (KSW), spontaneously fermented by lactic acid bacteria, as a therapeutic agent among Ghanaian children with diarrhoea	No effects of the intervention were found with respect to stool frequency, stool consistency and duration of diarrhoea; but KSW was associated with greater reported well-being 14 days after the start of the intervention	A preventing effect of KSW on antibiotic-associated diarrhoea which could help reduce persistent diarrhoea
PAPERS ON NEONATAL HEALTH					
1.	Chandramohan et al., 2005 [133]	Implemented an intervention in CH	The effects of intermittent preventive treatment for malaria in infants with sulfadoxine-pyrimethamine in an area of intense, seasonal transmission	Intermittent preventive treatment for malaria with sulfadoxine-pyrimethamine proved effective in reducing malaria and anaemia in infants	There is concern about the possibility of a rebound in the incidence of malaria in the second year of life despite its effectiveness in the previous year
2.	Edmond et al., 2008 [134]	Evaluated CH intervention, which is already operational	Diagnostic accuracy of a verbal autopsy (VA) tool in ascertaining the causes of stillbirths and neonatal deaths	The VA performance for stillbirth diagnoses is poor; accuracy was higher for intrapartum obstetric complications and antepartum maternal disease. For neonatal deaths, sensitivity was > 60% for all major causes Overall, VA diagnostic accuracy was higher than expected.	Further modifications are needed in the use of WHO VA in routine child health programmes; there is also the need to access the diagnostic accuracy of the VA tool in other regions and in multicentre studies
4.	Bryce et al., 2010 [135]	Evaluated a CH intervention already operational	Analysed how the Lives Saved Tool (LiST) was used as part of an early assessment of the expected impact of MCH intervention plans	Compared to 2006 levels, under-5 mortality could be reduced by at least 20% by 2011 by achieving national coverage targets for just four or five high-impact interventions	The feasibility and usefulness of LiST as part of the programme planning process at the community levels requires further experience
5.	Oduro et al., 2012 [136]	Health and demographic surveillance system profile	The activities and potential of the NHDSS for collaborative research are described	Using the NHDSS data, the attainment of the child survival Millennium Development Goals has been rapid with huge decline in maternal mortality ratio and the impact of immunisation on the relationship between poverty and child survival in the operational area	NHDSS has been designed to provide an efficient platform for evaluating health and social interventions
6.	Hulton et al., 2014 [137]	Use of evidence in MCH policy	Introduces the Evidence for Action (E4A) programme, the rationale and its effectiveness in initial findings across six E4A countries	There were inadequacies in using data for decision-making and in political will for MNH for all E4A countries; gaps in data access and information were key drawbacks to decision-making in all six countries	Given that this approach is effective in dealing with the deficiencies responsible for poor quality of care, then others can build on this to make future investment in MNH more cost-effective
7.	Hill et al., 2008 [138]	Use of knowledge in CH intervention	Described how an integrated home visit intervention for newborns in Ghana was designed	Identified community entry activities in mobilising community support for the intervention, to encourage self-identification of pregnant and delivered women and to motivate the volunteer through community recognition	Formative research is an important stage in helping to ensure the development of an effective, appropriate and sustainable intervention
8.	Moyer et al., 2013 [139]	Analysed an MCH intervention already in practice	Determined the types of access to care most strongly associated with facility-based delivery among women	Affordability was the strongest determinant linked to delivery location; social access variables, including needing permission to seek healthcare and not being involved in decisions regarding healthcare contributed in reducing the possibility of facility-based delivery when examined individually	Even among women with health insurance, affordability remains an important determinant of facility delivery; however, affordability was an important determinant of facility delivery in Ghana, even among women with health insurance, but social access variables had a mediating role

**Table 5:** Profile and characteristics of papers on scaling up of reproductive and child health interventions in Ghana

S/No	Author/year of publication/reference	Category of intervention	Health issue of intervention	Evidence generated	Policy-relevant conclusion
1.	Kapungu et al., 2013 [140]	Evaluation of phase 1 of a maternal and child health (MCH) intervention already operational	Operations research study, designed to reduce postpartum haemorrhage-related morbidity and mortality	Overall, 96% of deliveries resulted in healthy outcomes for the mother, with only 4.0% of births having complications	The initial work carried out in Phase 1 of the study is vital in guiding misoprostol distribution in Phase 2 although challenges exist
2.	Witter et al., 2009 [108]	Evaluates maternal healthcare (MHC) intervention already operational	National delivery exemption policy for free MHC	Delivery exemptions can be effective and cost-effective, and despite being universal in application, it can benefit the poor; however, there is the need for adequate funding and strong institutional ownership	Appropriate and effective implementation of the free MHC policy is key if it is expected to result in reduced mortality for mothers and babies
3.	Twum-Danso et al., 2014 [123]	Evaluated MHC intervention already in operational	Test the feasibility and effectiveness of the new early post-natal care (PNC) policy and its subsequent scale-up throughout northern Ghana	There is a slower increase in skilled delivery over a longer period of time; the early PNC policy was scaled up over the subsequent 2 years to 576 health facilities in all 38 districts of northern Ghana	The study provides a model for improving the implementation of other national health policies to accelerate the achievement of the Millennium Development Goals in Ghana and other resource-poor countries
4.	Singh et al., 2013 [130]	Impact evaluation of MCH intervention already operational	Evaluate the influence of the early phase of Project Fives Alive!, a national child survival improvement project, on key MCH outcomes	There was an association between the early pregnancy identification change categories with increased skilled delivery; additionally, a greater number of change categories tested was associated with increased skilled delivery	The quality improvement approach of testing and implementing simple and low cost locally inspired changes has the potential to lead to improved health outcomes at scale both in Ghana and other low- and middle-income countries
5	de Savigny et al., 2012 [141]	Adoption of innovation in the health system	Evaluated the introduction of vouchers for malaria prevention in Ghana and Tanzania	Investment in long-term, managed stakeholder engagement throughout the design and implementation stages of new complex health interventions appears to be critical for ownership and sustained integration of the intervention in the system	Contextual requirements for the success of an intervention should be considered before an intervention is picked from one context and piloted in another
6.	Philips et al., 2007 [142]	Evaluation of a scaled-up intervention	Used research to guide the development and scaling up of community-based health and family planning programmes	The process concluded with research-guided programme expansion, with each stage associated with shifts in generations of questions, mechanisms and outcomes as the process unfolded	Large-scale health systems development was achieved
7.	Awoonor-Williams et al., 2004 [143]	Evidence-based innovation and health-sector reform gap	Bridging the gap between evidence-based innovation and national health-sector reform	The favourable effect of the community-based health planning and services intervention on family planning and safe-motherhood indicators is suggestive that innovations such as the Navrongo experiment is transferable to impoverished rural settings elsewhere	The results confirm the need for strategies to bridge the gap between Navrongo evidence-based innovation and national health-sector reform
8.	Awoonor-Williams et al., 2013 [144]	Evaluated the impact of a MCH intervention	Described the Ghana Essential Health Intervention Project (GEHIP), a plausibility trial of strategies for strengthening Community Health Planning and Services, especially in the areas of maternal and newborn health	GEHIP improves the Community Health Planning and Services model in various ways	GEHIP is expected to contribute to national health policy, planning and resource allocation that will be needed to accelerate progress with the Millennium Development Goals
9.	Hill et al., 2010 [145]	Evaluated CH intervention	collected data on thermal care practices in rural Ghana to inform the design of a community newborn intervention	Respondents knew that keeping the baby warm was essential for health, but 71% of babies born at home had delayed drying, 79% delayed wrapping, 93% early bathing and 10% were placed skin-to-skin	Thermal care is a key component of community newborn interventions, the design of which should be based on an understanding of current behaviours and beliefs
10.	Awoonor-Williams et al., 2013 [146]	Lessons learnt from a scaled-up intervention	Strengthening of health systems related to maternal health	That community-based care could reduce childhood mortality by half in only 3 years	Key scaling-up lessons: (1) place nurses in home districts but not home villages, (2) adapt uniquely to each district, (3) mobilise local resources, (4) develop a shared project vision, and (5) conduct ‘exchanges’ so that staff who are initiating operations can observe the model working in another setting, pilot the approach locally and expand based on lessons learned

In the second stage, a draft report based on data collected in the first stage was presented to a meeting of 36 participants drawn from Ghana’s health sector (i.e. mainly policy-makers/managers/senior officials within the MOH and related agencies, local and international NGOs in the health sector, donors and academia) and working in RCH for comments. The comments provided were used to amend the draft report. In addition to the comments, a questionnaire was administered to participants of the stakeholder meeting, to captured respondent data in relation to official designation attributes; knowledge and application of information communication technology; knowledge of the policy-making process; capacity to use evidence; knowledge of policy and policy-making processes related to MNCH; acquisition of research evidence relevant to MNCH; assessment of the validity, quality and application of research evidence relevant to MNCH; ability to adapt formats of research results to provide information useful to decision-makers in MNCH; and application of evidence in decision-making relevant to MNCH.

A total of 15 participants (GHS *n* = 7, MOH *n* = 1, NGOs *n* = 4, Donors and Academia *n* = 4) responded to the questionnaire. Besides the questionnaire, we solicited and incorporated the views of the 36 participants on what they believe promotes the use of evidence in their workplace through group discussions. Specifically, participants deliberated on issues related to aptitudes, skills, institutional environment, platforms/mechanisms, sources of evidence, nature of evidence, opportunity for the use of evidence and support needed to use evidence for policy formulation. Although participants in the group discussion and respondents to the questionnaire were in the stakeholder meeting as representatives of their respective organisations, their participation was based on their consent and not on compulsion. Data from the questionnaire and group discussions was analysed and reflected in the draft report. It is important to emphasise that data collected was analysed and presented along the themes/components of knowledge transfer and research utilisation framework presented in Fig. 1.

## Results

In this section, we present the findings of the study structured around the components of the knowledge transfer and research utilisation framework.

### Problem identification and communication

In agreement with the core issues identified under this component, we sought to elucidate, from the data collected, the procedure used to identify RCH research needs and how those needs were communicated to researchers. Discussions with key managers within the health sector, as well as informal networks of individuals who have once worked in policy positions in the health sector, suggest that there is currently no institutional structure either at the level of the MOH or GHS that identifies research needs of units and directorates and channels them to researchers either within the health sector or outside of it.

This notwithstanding, a few units collate their research needs and incorporate this information into their annual plans to be performed when funds are available either through Government of Ghana budget or from donor funds. Within the RCH unit of the GHS, for example, the practice has been to aggregate research needs and incorporate these as part of the programme of work for the year, with the actual research carried out when funds become available either through the Government of Ghana budget or donor-funded programmes. Besides the above, there are also mechanisms that make it possible for research needs of the sector to be communicated to donors so that funds are made available to undertake such research or incorporated into the objectives of existing research funded by donors.

The mixed picture given by respondents interviewed and participants of the group discussion seems to be confirmed by the answers to the questions exploring the (1) existence of a forum or process to coordinate the setting of health research priorities or the (2) alignment of performance incentives to activities encouraging use of research evidence, in the survey carried out during the stakeholder meeting in Accra. The results suggest that 71.43% of respondents of the first question agree to the existence of a forum/process used to coordinate the setting up of research priorities in the health sector. On the contrary, the results of the second question suggest that approximately 60% of respondents believe that their management’s participation in fora that discusses research evidence related to organisational goals is inadequate.

However, it is important to emphasise that the lack of a structured institutional mechanism within the health sector to identify research needs and communicate these to researchers does not in any way mean that policy-makers are not interested in the use of research evidence to inform policy-making. However, the findings suggest that the challenge is rather the lack of local funds to carry out research. The findings further suggest that a greater proportion of research in Ghana’s health sector is funded by donors, whose objectives may not always coincide with the goals of domestic policy-makers. Thus, policy-makers are less likely to plan for their research needs given that they are less likely to receive funds domestically to carry out the research.

### Knowledge/research development and selection

#### Production, synthesis and adaptation of knowledge

In line with the conceptual framework, this section presents evidence that can be reasonably used to examine the capacity of the health sector to generate, synthesise and adapt RCH evidence to inform RCH policy.

Information provided on the website of the GHS, in addition to discussions with key managers within the GHS, suggest that there are currently formal structures in place to ensure a coherent approach to evidence gathering and synthesis. The GHS has a Research and Development Division headed by a director. The division has three research centres (NHRC, KHRC and DHRC), in addition to three other departments responsible for (1) documentation, dissemination and advocacy, (2) ethics and research management and (3) research. As captured on the GHS website, “*the key mandate of the RDD* [Research and Development Division] *is to generate information through relevant research to strengthen decision making, set health priorities, efficient resource allocation, and inform health interventions planning and implementation in order to deliver better health services to improve health status of the Ghanaian population*” [42].

To confirm that the information above, as captured on the GHS website, is operational, a review of the websites of the three research centres was performed. The results from the review suggest extremely impressive activity in terms of completed projects, on-going projects and new projects funded by internationally reputable funding organisations such as WHO, International Labour Organisation (ILO), Bill and Melinda Gates Foundation, Rockefeller Foundation, Pfizer Corporation, Volkswagen Foundation, National Institute of Health, World Bank, West African Health Organization (WAHO), and other bilateral and multilateral donors. In addition to these projects, the three centres have impressive outputs in the form of peer-reviewed scientific publications from completed projects.

For example, the information on the website of the DHRC showed 6 completed projects, 3 on-going projects and 29 peer-reviewed scientific publications [43]. In Dodowa, 4 out of a total of 9 projects and 5 out of a total of 29 scientific publications were on RCH. The NHRC, on the other hand, had 9 new projects, 16 on-going projects, 51 completed projects and 164 peer-reviewed scientific publications [44]. Out of a total of 73 projects in Navrongo, 17 were on RCH, with 29 out of a total of 164 scientific publications being on RCH. The KHRC had 31 completed projects and 6 on-going projects [45]. Most importantly, issues on MNCH featured strongly in the projects executed and scientific publications arising from the work of all the three research centres.

Besides the work of the research centres, the capacity of actors within GHS (staff and their collaborators) to produce good quality scientific evidence on MNCH was also examined. The results (Table 3) suggest that personnel at different levels of the GHS hierarchy are actively involved (i.e. either solely or in collaboration with others) in the production of scientific research evidence on RCH. In addition, evidence that suggests the availability of RCH research, either synthesised or in raw form, that could potentially and readily be used in the formulation of RCH policy was also examined. Overall, 28 scientific peer-reviewed publications were examined (Table 4), including 11 on maternal health, 9 on child health and 8 on neonatal health. Out of the 28 papers, 2 were systematic reviews of the existing literature, 3 on use of evidence in policy-making and 23 on the evaluation of interventions already in operation. This is indicative of the existence of extensive evidence on RCH produced outside of the GHS but available to policy-makers within the GHS.

The output of the research centres is also augmented by a strong health information management system, which collects, processes and stores data from different clinical and non-clinical institutions within the GHS. Discussions with actors within and outside of the GHS indicate that the output of the health information management system constitutes strategic evidence (inputs) into different policies, including RCH. The health information management system is equally augmented by data from several demographic surveillance systems, in addition to data from nationally representative surveys such as the GDHS and the Ghana Living Standards Survey, conducted every 5 years. Mostly, data from the three sources mentioned above are processed and presented in a format that can easily be used by policy-makers.

Clearly, the sources above point to the fact that the health sector in Ghana has enormous capacity both at the individual and organisational level to produce the much-needed evidence for policy formulation. This view is equally supported by results of responses to the questionnaire administered to participants of the Accra stakeholder meeting. The majority of respondents (as per their answers to different questions regarding the capacity of individuals within the GHS and the GHS as an institution to perform good quality research) believe that the GHS and its staff have adequate capacity to produce good quality evidence to aid policy-making.

#### Characteristics of knowledge to be transferred

This sub-section examines issues related to (1) the relative advantage and complexity of the knowledge to be transferred, (2) the compatibility of the knowledge to be transferred with existing beliefs and organisational norms, and (3) how the knowledge to be transferred is aligned to the needs of policy-makers.

Most of the research outputs found on the websites of the three research institutions are in the form of peer-reviewed research papers instead of alternative forms, such as policy briefs, that can be easily used by policy-makers. Although respondents to the questionnaire suggest that knowledge of policy briefs and how to use them is widespread in Ghana’s health sector, the evidence from the study suggests that RCH-related researchers in Ghana hardly use such means to disseminate their findings.

Although some respondents during the group discussion argued that, generally, public institutions in Ghana do not have the culture of evidence-based policy-making, this was not seen to be the case in the health sector. The difference may be due to the fact that the health sector has relatively better qualified staff who are also more experienced in research compared to other sectors in Ghana. Thus, the use of research evidence in policy-making may not be foreign to such actors. This assertion is equally confirmed by the results from the questionnaire. However, what may constitute a challenge is the extent to which the available evidence is aligned to the needs of policy-makers. As earlier on indicated, and as confirmed by the results of the questionnaire, a larger proportion of research in the health sector in general, and on RCH in particular, are mostly funded by donors who often have their own objectives, which may be different from those of policy-makers. Thus, besides a few circumstances where users of evidence collaborate with donors to conduct research, evidence produced from on-going research may not be aligned to the evidence/knowledge needs of policy-makers. This therefore constraints on-going efforts to improve the use of evidence to inform policy.

### Analysis of context

This section deals with identified barriers and incentives to knowledge transfer at the individual, organisational and environment level. As is evident in the research output of the three research centres and the large presence of GHS staff in the RCH literature, the capacity of individuals within the health sector to produce, synthesise and potentially use research evidence in the formulation of RCH policies cannot be in doubt. In addition, the multiplicity of professionals (medical doctors, nurses, allied health professionals, academics, etc.) within the health sector creates internal competition with respect to knowledge production and synthesis at the individual level. It is important though to caution that the capacity to produce and use evidence at the individual level may not in itself translate into the actual use of evidence in policy formulation.

Besides the individual level capacity, the findings of the study also suggest that there are several institutional and environmental incentives that promote the use of evidence in policy formulation within Ghana’s health sector. These include the sector-wide performance assessment framework linked to a set of internationally acceptable indicators that are also evidence-based, the subscription of the government through the MOH to major health-related international policies and conventions that are based on research evidence. This is evidenced by the several guidelines and protocols developed to ensure that actions at lower levels are consistent with the policies, protocols and conventions subscribed to. In addition, the evidence-based nature of work in the health sector makes it an environment that generally has few barriers to the use of research evidence for policy-making. This notwithstanding, major constraints to the use of evidence in policy include (1) how to ensure that research in general, and for that matter health-related research, gets the right attention at the highest level of political and administrative leadership so as to attract the right level of funding, and (2) the lack of robust and comprehensive institutional structures that ensure that the numerous health sector-related research being performed (i.e. whether specifically related to RCH or other relevant health issues) is aligned to the needs of policy-makers. The current disconnect between knowledge production activities and the needs of policy-makers eventually affects the extent to which policy-makers make use available knowledge to inform policy formulation. This position is confirmed by the results of the survey (questionnaire) where respondents suggest that institutional level incentives for (1) carrying out research, (2) use of research evidence implementation committees, (3) capacity to present research evidence in concise and accessible languages, and (4) capacity to synthesise different research evidence that addresses a common problem into a single document that could be attractive to policy-makers are inadequate, albeit that the required capacity and willingness to carry out good quality research exist at the individual level.

### Knowledge transfer activities or interventions

This aspect of the knowledge transfer and utilisation framework looks at actual interventions put in place to facilitate/ensure the transfer of knowledge/evidence for the purposes of policy-making. In the context of Ghana’s health sector, the most common intervention is post research dissemination workshops/conferences. In few instances, targeted policy briefs from completed research are disseminated to key stakeholders. However, as already indicated based on evidence from the three research centres, the use of policy briefs as a tool for ensuring the uptake of research evidence into policy in general and RCH in particular is rather scarce. The evidence gathered also suggests that advocacy by researchers as well as dialogue between donors and policy-makers constitute some of the channels via which research evidence on some key policy issues are discussed and consensus is reached on the possibility of adopting such evidence to aid policy formulation.

### Knowledge/research utilisation

This component of the knowledge transfer process looks at the actual use of available evidence in the formulation of policies. Following from the evidence gathered, we examined the level and extent to which the extensive evidence available, both within and outside of the GHS, is used in the formulation of policies and guidelines. Thus, 8 policy documents (6 on maternal health, 1 on child health and 1 on newborn health; Table 1) were accessed and reviewed with the aim of identifying whether the contents were informed by existing scientific evidence. The findings indicated that 3 out of the 8 policy documents reviewed used an evidence-based approach (i.e. either through citation of scientific research publications, synthesis of research evidence from the academic literature or adaptation of benchmarks from key stakeholder organisations such as WHO) in developing the document. In addition to the policies, 15 standard protocols and practice guidelines (Table 2) covering maternal and adolescent health (*n* = 11), neonatal and child health (*n* = 1), general infection prevention and control (*n* = 1) and malaria prevention (*n* = 2) were also reviewed. Out of the 15 protocols and practice guidelines, 5 were evidence informed. Surprisingly, however, the policy documents and practice guidelines that suggested the use of scientific evidence did not explain the evidence generation processes and how the evidence was used in the policy/practice guidelines. Nevertheless, it is clear that the 8 policy documents and 15 protocols and practice guidelines were all developed through a consultative and participatory approach mostly involving stakeholders and professionals knowledgeable in the subject area. It is also important to state that discussions with key managers within the RCH division of the GHS indicated that almost all the standard protocols and practice guidelines in use are adaptations from regulators or key global institutions (WHO, UNICEF, UNFPA World Bank, etc.), which are known to rely on scientific evidence in the production of such guidelines.

In addition to the review of the policies and practice guidelines, another channel through which knowledge translation may occur (i.e. scaling up of interventions based on evidence from a pilot phase) [46, 47] was also examined. The results show (Table 5) that 10 important interventions were scaled-up based on evidence from the pilot/experiment phase. A key intervention in this direction is the Navrongo experiment (i.e. the Community Health Planning and Services (CHPS) concept). Lessons learnt from the Navrongo experiment were crucial to the scaling up of the CHPS concept, which currently constitutes a key ‘Safe Motherhood’ strategy in Ghana. There is also the possibility of the scaling up of capitation as a payment method across Ghana after initial piloting in the Ashanti region of the country. There is also evidence to the effect that the decision to discontinue vitamin A in pregnant women was based on research that found that it did not have any significant positive effect on pregnant women [48]. Again, it is on record that the Emergency Obstetric, Maternal and New Born Care study in Ghana [49] informed GHS’ adoption of the maternal acceleration fund policy to facilitate MNCH in 2011. Additionally, evidence from the EMBRACE study is currently being used by the GHS to adopt a special maternal card to help track the progress of pregnant women so that they can adhere to their continuum of care [50]. Information from the DHRC also suggests the use of research evidence to support health policy. For example, project briefs from the DHRC show policy impact in terms of level of policy-making, type of policy, nature of policy impact, policy networks, political capital and inclusion in policy documents.

From the above, one can argue that, although a structured and robust organisational mechanism to ensure the use of scientific research evidence to inform policy formulation does not exist, actors within the health sector are relying on existing, albeit weak, institutional platforms to transfer knowledge gained from research to policy and practice.

## Discussion

The information extracted in this study is herein used to answer the key question of whether Ghana’s health sector has institutional structures or mechanisms in place for the production and use of evidence to inform policy formulation in general and RCH policies in particular.

The findings above suggest that there are currently organisational-wide structures in place for the production of RCH evidence. This is based on the fact that the GHS has a well-functioning and extremely active research and development division, as is evident in the number of projects and peer-reviewed scientific research publications completed. The fact that most of the research projects carried out by the research centres are funded by internationally reputable funding organisations such WHO, ILO, Bill and Melinda Gates Foundation, Rockefeller Foundation, Pfizer Corporation, Volkswagen Foundation, National Institute of Health, World Bank, WAHO, and other bilateral and multilateral donors, speaks to the quality of their research output. It is important to emphasise that quality, as used in this context, is mainly in reference to the rigorous nature of the evidence produced as opposed to coverage (i.e. relevance to policy-makers or population health needs). The rigour argument is based on the fact that funding organisations, such those indicated above, will normally ensure that research funded by them follow very rigorous standards.

Besides the output of the research centres, the findings indicate that staff from within the GHS are highly active contributors to the RCH literature in Ghana. Again, the existence of organisational and national level data repositories that can easily be accessed and used by policy-makers to aid policy formulation is ample testimony of the existence of high quality scientific research evidence that can be used to inform policy formulation. Additionally, the findings suggest that both GHS staff and other external collaborators perform scientific research within the GHS. Thus, the findings indicate to a certain systematic approach at the organisational level to enhance the production of evidence through scientific research.

The existing institutional arrangements for the production of evidence may, in some form, create opportunities for the use of evidence produced to inform policy. For example, there is evidence in the existing knowledge transfer and utilisation literature [41] that suggests that policy-makers (1) are more likely to trust evidence produced by their colleagues and therefore use such evidence to inform policy, (2) use evidence from research that involves them from planning to dissemination, (3) use evidence to inform policy if there is a window of political opportunity to do so, and (4) are more likely to use evidence to inform policy if they have a better link with the producers of evidence. Juxtaposing the results of the paper with prescriptions from the literature as indicated above, one would expect that existing GHS structures that promote the production of evidence (activities of the research centres, individuals either alone or in collaboration with others outside of the sector) by actors within the health sector may mean better credibility for such evidence and, by extension, the possibility that it will be used to inform policy. Secondly, the fact that these research centres/individuals are part of the GHS should ordinarily make it easy for those in charge of policy to have access to the producers of evidence, thereby enhancing the plausibility that available evidence will be used to inform policy formulation.

On the contrary, the results paint a mixed picture. One set of evidence indicates that systematic organisational structures as well as individual capacity that makes it relatively easy for policy-makers to use available evidence in the formulation of RCH policies exist. For example, project reports that capture the details (level of policy-making, type of policy, nature of policy impact, policy networks, political capital and inclusion in policy documents) of how findings of executed projects have been used in the formulation of policies is instructive. The fact that almost all the standard protocols and practice guidelines are adaptations from benchmark documents issued by regulators or key institutions such as WHO, UNICEF, UNFPA and the World Bank, which rely highly on scientific evidence in the production of such guidelines, is also important. Thus, modelling RCH protocols and guidelines around documents of such institutions suggests that such protocols and practice guidelines are informed by evidence.

Further, evidence on the scaling up of RCH interventions based on lessons learnt from the pilot phase of most of these projects is ample testimony that scientific research evidence gathered from project execution constitutes a relevant input to policy formulation and implementation. As already indicated, the scaling up of the Navrongo experiment (i.e. the CHPS concept) has had and will continue to have a profound impact on the nature and type of strategies adopted to improve RCH outcomes in Ghana. The possibility of scaling up capitation as a payment mechanism for Ghana’s social health insurance from one region/province to national coverage is also important in this direction. Additionally, the referencing of scientific research papers in the bibliography section of some of the policies, standard protocols and practice guidelines is suggestive that findings from such papers constituted a good piece of evidence used in the formulation of the respective policies. On account of this evidence, one can suggest that capacity exists within the GHS that makes it possible to use available scientific research evidence to aid the formulation and implementation of RCH policies.

Nevertheless, there are findings from the study that equally suggest the existence of constraints that can compromise the ability of the GHS to use existing evidence to inform RCH policy. For instance, several of the policy documents and standard protocols and practice guidelines did not have any scientific research publication acknowledged in the list of references. For example, only 3 out of the 8 policy documents reviewed cited scientific research papers. In the case of the standard protocols and practice guidelines, only 5 out of the 15 documents reviewed cited the use of a scientific research paper. More importantly, those documents that cited scientific research papers did not explain the processes through which evidence emanating from these scientific papers was captured and used in either policy formulation or drafting of the standard protocols and practice guidelines. Equally important in this regard is the apparent lack of both physical and electronic evidence that shows that findings from the numerous completed projects as well as peer-reviewed publications have been converted to a form that can be easily used by policy-makers (e.g. policy briefs or policy summaries). Additionally, the divergence of donor priorities and those of policy-makers in terms of research may mean misalignment between evidence produced from research and information needed by policy-makers at a particular point in time. Thus, RCH evidence may be available (e.g. output of the research centres) and yet may not be useful for decision-making. It is important to caution that the policy-maker/donor divergence in research priorities may not necessarily mean a misalignment between research output of the research centres and population health needs, given that research undertaken by the research centres and individuals within the sector are all on issues of national relevance. Thus, the identified shortcomings should be viewed mainly as structural weaknesses within the MOH and its agencies, specifically the GHS, which constrain the use of evidence to inform policy formulation, and not necessarily as evidence to support the notion that policies and guidelines are not informed by existing scientific research evidence.

However, what seems to be clear from the evidence gathered so far with respect to the management of the knowledge transfer processes in Ghana’s health sector and, for that matter, RCH, is that apart from the actual knowledge production component, the rest of the process seems to be rather informal. For example, the key components of problem identification and communication, implementation of interventions and actual use of evidence do not follow any structured institutional system. Additionally, funding for research is not centrally coordinated but mostly through an arrangement between individuals or clusters of researchers within the health system and donors. It is important to emphasise that these weaknesses have collective negative implications for establishing an appropriate monitoring and evaluation framework to evaluate the knowledge production process and how it is impacting policy formulation. It is therefore not surprising that key performance indicators used to evaluate sector-wide performance in Ghana do not capture knowledge production and transfer as an activity of interest to monitor [23, 24].

### Strength, weaknesses, opportunities and threats

A careful examination of the findings suggest that Ghana, compared to many sub-Saharan African countries, has made progress in producing evidence and using this to inform policy formulation. It is therefore important that the strengths of the existing system are highlighted and that appropriate measures are instituted to deal with inherent weaknesses and threats so as to take advantage of current and future opportunities to improve existing knowledge transfer processes in the health sector.

### Strengths and weaknesses

The findings are suggestive that, within Ghana’s health sector and particularly in the RCH division of the GHS, policy formulation is evidenced based. There is also enough evidence to suggest that the GHS has organisational level structures in place (the three research centres) to aid the production of the required evidence to inform policy formulation. Also important is the fact that the GHS has a critical mass of researchers who are extremely active in contributing to the production of scientific knowledge in RCH. As earlier indicated, formal and informal structures that drive organisational learning and norms that value the importance of evidence in decision-making [15, 19] are key to the use of evidence in policy-making. Thus, one can argue that the systems currently in place in the GHS, though not the very best, constitute good organisational capabilities and strengths that could be leveraged upon to further improve the capacity of the GHS in general, and the RCH division in particular, to produce and use scientific research evidence in the formulation of RCH policies and guidelines. Additionally, the fact that existing norms and beliefs within the health sector are consistent with knowledge production and transfer is important for improving evidence-informed policy and practice.

The ability of researchers to summarise very complicated scientific language into simpler but easy and ready to use material by policy-makers is seen as a key facilitator of research uptake into policy [36]. In this context, one can argue that the general inability to convert most of the several scientific publications by the three research centres into policy-friendly summaries or briefs that can easily be used by policy-makers, coupled with the absence of appropriate scientific references in most of the policy documents and practice guidelines, is indicative of internal organisational weaknesses. These weaknesses refer to the absence of systematic structures that ensure that best practices are defined and followed. Given that the standard protocols and practice guidelines are adapted as indicated above, the main documents on which they are based should be adequately cited and the evidence-based processes from which the main findings were synthesised should be transparently acknowledged and referred to in the new document.

A familiar argument within the knowledge transfer and research utilisation literature has been the need to bridge the cultural and institutional gap between researchers and policy-makers, given that it constitutes a major barrier to research uptake in policy formulation [51–53]. Thus, the absence of a central structure from within the health sector to coordinate key knowledge production and transfer actors (policy-makers, researchers and donors) as well as the absence of a structured approach to define research needs and linking these to ongoing research, constitute a major weakness in the knowledge production and utilisation chain. On the basis of the above and in line with Henkel [54], we argue that policy-makers within the MOH and its agencies may need to work hard not only to identify problems but also to identify research that may help solve existing problems in addition to using the research output. Further, the fact that knowledge utilisation processes are not transparently defined and incorporated into organisational systems within the sector is also problematic. It is important to highlight that the absence of an effective monitoring and evaluation function for knowledge production and, most importantly, for transfer and utilisation constitutes a system weakness.

### Opportunities and threats

Outside of the internal structures there are clear opportunities that can be exploited by the GHS to improve on its uptake of scientific research evidence as inputs to the policy formulation process. For example, the extensive network of collaborations with researchers outside of the GHS as well as access to major financiers (WHO, ILO, Bill and Melinda Gates Foundation, Rockefeller Foundation, Pfizer Corporation, Volkswagen Foundation, National Institute of Health, World Bank, WAHO and other bilateral and multilateral donors) of scientific research are all great opportunities that can be exploited to improve internal structures for producing and using good quality evidence to inform policy formulation and implementation. It is however important to emphasise that, to be able to take advantage of the opportunities enumerated, a major threat that ought to be managed well is the issue of funding. Issues around the nature and source of funding, as well as the coordination and management of such funding, are crucial. The fact that a larger proportion of funding for research in the health sector comes from outside the health sector budget is a threat to implementing any robust central coordinating mechanism for knowledge production and transfer.

It is important that as Ghana’s strengths and opportunities for knowledge production and transfer are highlighted, effective and immediate steps are taken to address inherent weaknesses and threats to ensure that it improves on the current system of producing evidence and using it to inform the formulation of RCH policies. As clearly articulated by the participants of the Accra stakeholder meeting, improving the knowledge transfer process is a collective task that must involve all stakeholders (key among them being researchers, policy-makers and donors) with the health sector taking the lead.

## Conclusion

Issues of RCH and MNCH are important areas in the strengthening health systems in developed and developing countries alike. In developing countries such as Ghana, the issue is even more critical given that health systems are generally weak and have low levels of investment and poor RCH indicators, especially during the 1990s. However, the implementation of pragmatic policies and commitment of health sector managers over almost two decades has ‘paid off’ in terms of tremendous improvement in the state of RCH.

Nevertheless, Ghana still has challenges in RCH and further efforts to improve RCH policy-making processes are likely to yield even better results. It is therefore important that the MOH and its agencies, especially the GHS, take advantage of its current strengths and opportunities (strong internal knowledge production capacity, access to knowledge production networks outside of the health sector, a positive environment for the promotion of evidence-informed policy, access to major donors with the resources to fund good quality research, etc.) to confront and address the structural and organisational weaknesses inherent in the process of translating research evidence to evidenced-informed policy. Specifically, there should be deliberate efforts to mainstream research in the health sector so that an appropriate budget can be allocated to fund research. The mainstreaming of research within the sector could also help resolve the issue of the establishment of research priorities and their coordination between researchers and policy-makers.

## Abbreviations

CHPS: Community Health Planning and Services
DHRC: Dodowa Health Research Centre
EBM: Evidence-based medicine
GDHS: Ghana Demographic and Health Survey
GDP: Gross Domestic Product
HE: Health expenditure
ILO: International Labour Organisation
KHRC: Kintampo Health Research Centre
MNCH: Maternal, newborn and child health
MOH: Ministry of Health
NHRC: Navrongo Health Research Centre
POW: Programme of Works
RCH: Reproductive and child health
WAHO: West African Health Organization
